# Healthy Pills: A Physical Activity and Meditation Program to Enhance Mental Health and Well-Being in Spanish University Students

**DOI:** 10.3390/bs15040549

**Published:** 2025-04-18

**Authors:** Laura García-Pérez, Rosario Padial-Ruz, Mar Cepero-González, José Luis Ubago-Jiménez

**Affiliations:** Department of Didactics of Corporal Expression, Faculty of Education, University of Granada, 18071 Granada, Spain; rpadial@ugr.es (R.P.-R.); mcepero@ugr.es (M.C.-G.); jlubago@ugr.es (J.L.U.-J.)

**Keywords:** mental health, psychological well-being, physical activity, meditation, university students, intervention-based study

## Abstract

(1) Background: University students’ mental health (MH) is in crisis due to academic stress, lack of physical activity (PA), and low self-esteem. This study evaluated a 12-week PA and meditation intervention to enhance psychological well-being in Spanish university students. (2) Methods: A quasi-experimental design was used, with a non-randomized control group and pretest-posttest assessments. The study lasted 14 weeks (12 weeks of intervention and two for evaluations). Initially, 149 students were recruited, but the final sample included 136 (82 intervention, 54 control) due to attrition. Participants were selected through convenience sampling, respecting university-established groups. The intervention consisted of six PA sessions (aerobic, cardiovascular, and strength exercises) and six meditation sessions (yoga and mindfulness). Validated questionnaires assessed resilience, psychological distress, self-esteem, mood, personality traits, sedentary behavior, PA levels, and sleep duration. (3) Results: Significant improvements were found in resilience (*p* < 0.001), depression (*p* < 0.01), and sleep duration (*p* < 0.05), with greater mood benefits in men. No major changes were observed in other variables. (4) Conclusions: PA- and meditation-based interventions can improve students’ MH, particularly in key psychological aspects. Further research should explore long-term effects and refine strategies by distinguishing between preventive and therapeutic approaches.

## 1. Introduction

University students are particularly vulnerable to poor mental health (MH) due to transitional life changes, academic stress, and social or financial insecurity (Kang et al., 2020; Porru et al., 2021). According to the World Health Organization (WHO), mental health (MH) is not merely the absence of mental disorders but a state of well-being in which individuals realize their potential, cope with normal life stressors, and contribute productively to their communities (WHO, 2022). In this study, psychological well-being is understood as a multidimensional construct that encompasses personal resources such as resilience and self-esteem, emotional states such as mood and psychological distress, and individual differences reflected in personality traits. This integrative approach allows for a comprehensive understanding of MH that extends beyond the absence of clinical disorders. MH conditions account for 16% of the global disease burden among young people (WHO, 2022). These conditions typically emerge between the ages of 14 and 25, making secondary schools and universities key environments for MH promotion, prevention, and intervention (Solmi et al., 2022).

Within the framework of the World Mental Health International College Student (WMH-ICS) Project, MH has been examined in students from 19 universities across eight countries (Auerbach et al., 2018), revealing that 20–30% experience at least one mental disorder within a 12-month period, with depression and anxiety being the most prevalent (Ballester et al., 2020; Emmerton et al., 2024). Additional studies identify various contributing factors, including sleep quality (Park et al., 2020), self-esteem (Vall-Roqué et al., 2021), academic performance (Bruffaerts et al., 2018), diet (Bremner et al., 2020), and financial difficulties (Bøe et al., 2021). In Spain, a single epidemiological study has provided an overview of the most common mental disorders among university students, finding that 48% exhibit moderate to severe symptoms of depression and anxiety, while 22% report suicidal ideation. Other studies have also contributed valuable data, offering a broader and more detailed perspective on student MH by highlighting high prevalence rates of depression, anxiety, and stress in Spanish universities, as well as functional impairment associated with mental disorders in Mediterranean regions (Ballester et al., 2020; Blasco et al., 2019; Ramón-Arbués et al., 2020; Rodríguez-Rodríguez et al., 2022; Tomás-Gallego et al., 2024).

Physical activity (PA) has been widely recognized as an effective strategy for pre-venting and managing MH disorders, supported by strong empirical evidence demon-strating its capacity to enhance emotional well-being and reduce psychological distress (Remskar et al., 2024; Vella et al., 2023). Regular PA significantly improves psychological well-being by promoting endorphin release and lowering cortisol levels, a hormone associated with stress (Mahindru et al., 2023). Recent studies have documented significant re-ductions in symptoms of depression, anxiety, and stress, as well as improvements in mood following participation in PA programs (Chan et al., 2019; Singh et al., 2023). These benefits have been observed in various types of PA interventions, including aerobic capacity exercises (Song et al., 2021), resistance training (Gordon et al., 2018), and strength training (Barahona-Fuentes et al., 2021). This growing body of evidence has led international health organizations, such as the National Institute for Health and Care Excellence (2022) and the WHO (2024), to recommend PA as both a preventive and complementary approach to MH treatment. However, numerous studies have indicated the decline of PA levels among university students, thus making this population the focus for health interventions in many countries (Edelmann et al., 2022; Kumar et al., 2022). While at secondary school, there is a significant decline in students at the meeting level of the PA recommendations (Kann et al., 2018), and it carries over into higher education (American College Health Association, 2024). Despite the well-established benefits of PA on MH, university students are often not able to adhere to these recommendations, indicating the need for interventions that support greater levels of participation in PA and improved psychological functioning.

Meditation-based interventions (MIs), which promote mindfulness, tolerance, acceptance, and emotional regulation, have also been shown to enhance psychological fac-tors crucial for PA adherence and effectiveness (Schuman-Olivier et al., 2020). MIs activate the parasympathetic nervous system, reducing heart rate and blood pressure, relaxing muscles, and fostering a sense of calm and well-being (Khajuria et al., 2023). The combination of PA and MI presents a complementary approach in which meditation facilitates PA engagement by encouraging an open, non-judgmental attitude towards discomfort, while PA enhances motivation and a sense of achievement, essential for sustaining both practices over time (Remskar et al., 2024). This positive reinforcement cycle amplifies the MH benefits, particularly in university students, who report significant reductions in depression, anxiety, and stress when they comply with PA recommendations (Rodríguez-Romo et al., 2022; Herbert et al., 2020).

To maximize these benefits, interventions should incorporate behavioral activation strategies and psychoeducational support, fostering psychological resources such as motivation, self-regulation, and resilience, which further enhance PA’s impact on emotional well-being (Glowacki et al., 2017).

Despite strong evidence supporting PA’s role in mental well-being, research on structured PA interventions in university settings remains limited (Huang et al., 2024; Jeftic et al., 2023). However, integrating PA into higher education as a preventive measure is highly justified. Unlike traditional MH support services, PA is an accessible and cost-effective strategy that reduces barriers to engagement and is perceived by young people as a non-stigmatizing self-care practice with substantial health benefits (deJonge et al., 2020).

Considering this and based on the WHO’s (2022) definition of MH—which extends beyond the absence of mental illness to include psychological flourishing, such as resilience, self-esteem, and emotional well-being—this study aimed to evaluate the impact of a PA and MI on key variables related to psychological well-being. Specifically, it examined resilience, self-esteem, anxiety, stress, depression, personality traits, and mood in Spanish university students.

In addition, previous findings suggest that sex may moderate psychological responses to PA and meditation, particularly in terms of emotional regulation, resilience, and stress response (Agnieszka et al., 2020; Gök & Koğar, 2021; López-Madrigal et al., 2021). For example, men tend to report higher levels of PA and self-determined motivation (Espada et al., 2023; Romero-Parra et al., 2023), while women show greater openness to meditation and mindfulness practices, possibly due to a stronger focus on emotional well-being and self-reflection (Upchurch & Johnson, 2019). Considering these patterns, the present study also explored whether the intervention effects differed by sex.

It was hypothesized that students in the intervention group (IG) would exhibit significant improvements in resilience, self-esteem, and positive mood states, as well as significant reductions in psychological distress, including depression, anxiety, and stress, compared to the control group (CG). Moreover, it was expected that the impact of the intervention on these variables might differ by sex.

## 2. Materials and Methods

### 2.1. Design

A non-randomized quasi-experimental design was used, featuring a non-equivalent control group and pretest-posttest assessments. Convenience sampling was used, selecting pre-existing class groups within the Faculty of Education Sciences at the University of Granada. This decision was made due to the constraints of the educational/academic setting, where random allocation was not feasible due to logistical and operational limitations. Additionally, working with pre-existing groups optimized resources and ensured the feasibility of the intervention.

Although no prior sample size calculation was performed, the selected groups included all students enrolled in the Physical Education program of the Faculty of Education Sciences during the study period, making the sample representative of this academic population. This ensured the relevance of the findings within the academic context. Statistical analyses were conducted based on the final group sizes, allowing for the evaluation of significant differences across key variables.

Since the intervention involved practical components such as meditation and physical conditioning, complete blinding of participants and facilitators was not possible. However, several strategies were implemented to minimize the risk of bias: (1) data analysts and outcome assessors were blinded to group allocation, (2) baseline comparisons were conducted to verify initial homogeneity between groups, and (3) standardized protocols were applied during the implementation of intervention. These measures helped strengthen the internal validity of the study.

The study design is detailed in the protocol published by García-Pérez et al. (2024).

### 2.2. Study Population

**Figure 1:**
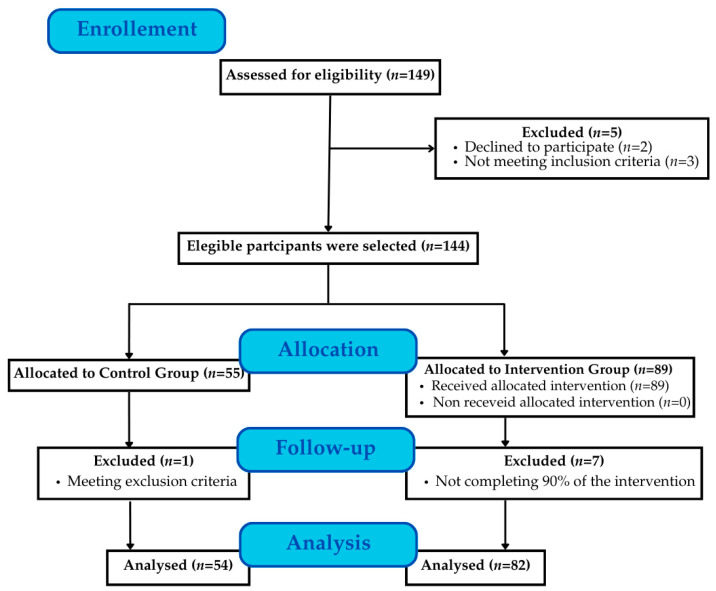
Flow of participants.

A total of 149 students from the Faculty of Education Sciences at the University of Granada (Andalusia, Spain) were assessed for eligibility and inclusion in the study (Figure 1). All participants were in their fourth year of the primary education degree, specializing in physical education, during the 2023/2024 academic year and followed a continuous assessment evaluation mode. Inclusion criteria were (1) being enrolled in the physical education specialization of the primary education degree program and (2) availability to attend at least 80% of the intervention sessions.

The exclusion criterion was the presence of extreme clinical MH states such as schizophrenia, bipolar disorder, severe depression, or severe anxiety. During screening, university students were asked if they had any of these conditions, and the students who answered in the affirmative to having them were excluded from participation. In cases where participants had medical or psychological records, these were reviewed to confirm the non-presence of such conditions. This stipulation was established so the impact of the intervention could be quantified within a population that had no serious underlying MH issues that would muddy the results. Additionally, a non-inclusion criterion was established: students who participated in the intervention but attended less than 90% of the sessions were excluded from the statistical analysis.

A total of 13 students were lost due to the following reasons: refusal to participate (*n* = 2), failure to meet inclusion criteria (*n* = 3), incomplete evaluations (*n* = 1), and attendance below 90% (*n* = 7). As a result, the final analysis included 136 participants, aged 21 to 25 years (21.70 ± 0.95). The IG comprised 82 students, while the CG included 54 students. The loss of participants did not significantly affect the study outcomes.

### 2.3. Intervention Structure

**Figure 2:**
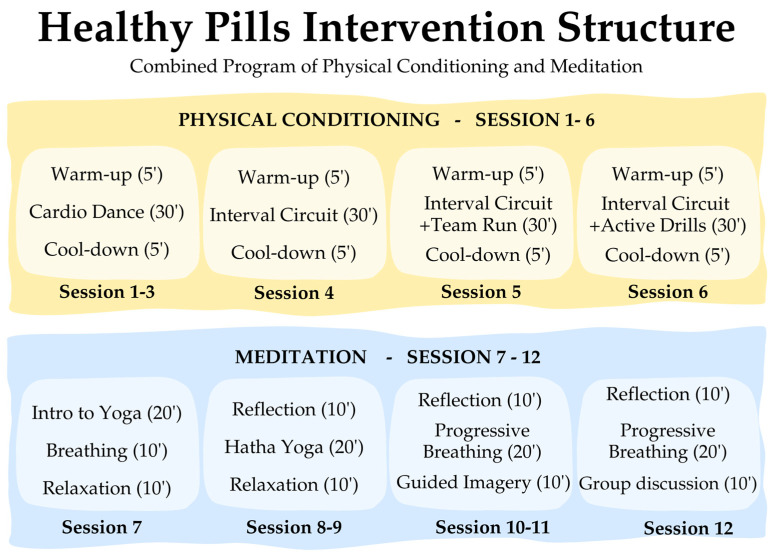
Summary of the intervention.

The “Healthy Pills” intervention was administered to the experimental group over 12 weeks, over three months, with weekly sessions lasting 40 min. The intervention was conducted from September to December 2023. Both the CG and IG completed the pretest one week before and the posttest one week after the intervention. The structure of the intervention is summarized in Figure 2 (while a more detailed version is available in Supplementary Materials, Figure S1).

The intervention was designed as a combined program including both PA and meditation strategies. This integrated format was selected in line with existing evidence supporting the complementary benefits of both components for psychological well-being (García-Pérez et al., in press). Moreover, the program was implemented within a university course, and logistical constraints prevented the formation of multiple intervention groups.

The program comprised two main blocks. The first block, “Physical Conditioning”, comprised six sessions focused on aerobic capacity, cardiovascular fitness, and muscular strength. Each session followed a structured format, including a warm-up, main training phase, and cool-down. Sessions 1–3 focused on progressive aerobic and cardiovascular activities, incorporating controlled movements that gradually increased in intensity and difficulty over the course of sessions 1–3. Sessions 4–6 introduced bodyweight strength training, structured as interval-based circuits targeting large muscle groups.

The second block, “Meditation”, also included six sessions, integrating breath-based meditation (mindfulness and internal gymnastics) and body-mind movement-based meditation (yoga). Each session followed a structured approach, including a theoretical introduction, guided practice, and a final relaxation phase. Sessions 7–9 were yoga-based, emphasizing physical, psychological, and spiritual integration, with reflections on inner balance. Sessions 10–12 focused on mindfulness and internal gymnastics, encouraging awareness of bodily sensations through progressive breathing techniques and deep visualization exercises.

**Figure 3:**
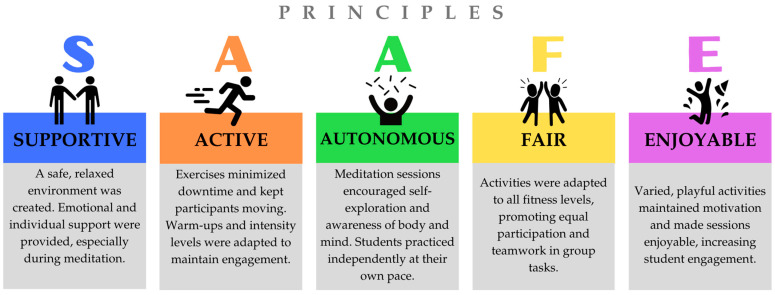
Principles of SAAFE.

The intervention followed the SAAFE principles, as outlined by the Priority Research Centre for Physical Activity and Nutrition at the University of Newcastle (Figure 3). Additionally, the Transparent Reporting of Evaluations with Nonrandomized Designs (TREND) guidelines were followed to ensure transparency and quality (Des Jarlais et al., 2004) (Supplementary Materials, Table S1). A fidelity analysis was conducted to confirm that the observed outcomes were attributable to the program, following the steps outlined by Nelson et al. (2012), which include defining the intervention, developing a measurement system, monitoring implementation, evaluating fidelity, and providing feedback for adjustments. Standardization and protocolization ensured that all components were implemented consistently and systematically.

The selection and training of facilitators was a key aspect, ensuring they understood the objectives and strategies and delivered the intervention accurately. Evaluation criteria included protocol adherence and participant responsiveness. Finally, continuous monitoring through observations and feedback allowed for necessary adjustments to maintain program integrity, effectiveness, and replicability.

### 2.4. Instruments

Sociodemographic data were collected through an ad hoc questionnaire, which included questions on sex, age, nationality, type of residence, receipt of financial aid during the academic year, academic performance, and smoking habits. The questionnaire was completed online using Google Forms. Additionally, the forms were filled out in the presence of the researchers to address any questions or issues that participants might have had during the data collection process, ensuring accurate and reliable responses.

#### 2.4.1. DASS-21

To assess psychological distress, the Depression, Anxiety, and Stress Scale-21 (DASS-21) (Lovibond & Lovibond, 1995) was used. This tool evaluates three core MH dimensions: depression, anxiety, and stress. The validated Spanish version (Daza et al., 2002) was employed, demonstrating strong validity and reliability in this study, with high internal consistency (α = 0.94; 95% CI: 0.92–0.95), as previously reported in similar research (Kavvadas et al., 2023). The questionnaire consists of 21 items, distributed across three subscales, with responses on a 4-point Likert scale, ranging from 0 (not at all) to 3 (applies a lot or most of the time). The final scores for each subscale are obtained by summing the respective item scores, with a total subscale score ranging from 0 to 21.

#### 2.4.2. Connor–Davidson Resilience Scale

The Connor–Davidson Resilience Scale (CD-RISC) (Connor & Davidson, 2003) was used to assess resilience. The validated Spanish version (Manzano-García & Ayala Calvo, 2013) has demonstrated strong reliability in university populations (Gras et al., 2019). In this study, Cronbach’s alpha was 0.85 (95% CI: 0.81–0.88), indicating high internal consistency and reliability. This 25-item scale is rated on a 5-point Likert scale (0 = not at all true; 4 = almost always true), with total scores ranging from 0 to 100, where higher scores indicate greater resilience.

#### 2.4.3. Self-Esteem Scale

Self-esteem was measured using an adapted and validated Spanish version of the Rosenberg Self-Esteem Scale (Rosenberg, 2015; Atienza et al., 2000), which has been widely applied in research with similar populations (Acosta-Gonzaga, 2023). The scale demonstrated high internal consistency (α = 0.84; 95% CI: 0.80–0.87), confirming its validity and reliability. The scale consists of 10 items, with five positively worded and five negatively worded statements to control for response bias. Items are rated on a 4-point scale (A–D), with positively worded items scored from 4 to 1 and negatively worded items scored from 1 to 4. The total score, obtained by summing all item scores, ranges from 10 to 40, where higher scores indicate greater self-esteem.

#### 2.4.4. Profile of Mood States

Mood states were assessed using the shortened version of the Profile of Mood States (POMS) questionnaire (29 items), validated in Spanish by Andrade et al. (2010). This version employs a 5-point Likert scale (0 = not at all; 4 = extremely) and measures five mood dimensions: one positive (Vigor) and four negative (Tension, Depression, Anger, and Fatigue). Subscale scores were obtained by summing item scores for each dimension. The questionnaire demonstrated high internal consistency (α = 0.82; 95% CI: 0.81–0.82) in this study and has been widely used in university research (Barney et al., 2022).

#### 2.4.5. Big-Five Inventory

The Big Five Inventory (BFI-44) (Benet-Martínez & John, 1998) was used to assess personality traits. This self-report questionnaire evaluates five core personality dimensions: Extraversion (8 items), Agreeableness (9 items), Conscientiousness (9 items), Neuroticism (8 items), and Openness to Experience (10 items). Participants responded on a 5-point Likert scale (1 = strongly disagree; 5 = strongly agree), with higher scores indicating stronger trait expression. In this study, the BFI-44 demonstrated high reliability and internal consistency (α = 0.73; 95% CI: 0.71–0.75) and has been extensively validated in university populations (Yang et al., 2022).

#### 2.4.6. International Physical Activity Questionnaire

The short version of the International Physical Activity Questionnaire (IPAQ-SF) (Craig et al., 2003) was used to assess overall physical activity (PA) levels. This questionnaire has demonstrated reliability coefficients exceeding 0.65 and a combined ρ = 0.76 (95% CI: 0.73–0.77), confirming its validity in similar studies (Li & Li, 2022).

The IPAQ-SF total PA score was calculated by summing the reported time spent on different PA intensities, resulting in a combined PA score expressed in MET-minutes per week (IPAQ Committee, 2005). Additionally, the questionnaire included a specific item on sedentary behavior, measuring the average daily sitting time in a typical week.

#### 2.4.7. Sleep Assessment

Sleep patterns were evaluated using self-reported questions regarding participants’ sleep habits. Participants reported their average sleep duration on weekdays and weekends, which were then combined to calculate a weekly average sleep duration, providing a representative estimate of total sleep time per week.

### 2.5. Ethical Clearance

All participants voluntarily took part in the study. University students provided written informed consent for both participation and the use of their images, including photographs and videos recorded during the intervention. Participants were assured of anonymity, voluntary participation, and that data would be processed exclusively for scientific purposes, in compliance with the General Data Protection Regulation (EU) 2016/679 (GDPR). Each participant was assigned a coded identifier to facilitate tracking throughout the intervention.

This study was approved by the Department of the University of Granada and the Human Research Ethics Committee of the University of Granada, under registration number 3678/CEIH/2023. The research groups HUM238 and HUM727 from the University of Granada supervised the study.

### 2.6. Statistical Analysis

Descriptive statistics (means, standard deviations, and percentages) were calculated for all variables. Normality was assessed using the Kolmogorov–Smirnov test with Lilliefors correction, while homoscedasticity was evaluated using Levene’s test. Since the variables did not follow a normal distribution, the Mann-Whitney U test was used to compare baseline characteristics between groups. The unit of analysis in this study was the group level, which coincided with the unit of assignment, as the intervention was implemented within pre-existing class groups. Consequently, the analysis compared these groups as independent units rather than individual participants. Within-group significant differences were analyzed using the Wilcoxon signed-rank test for paired samples. All data were analyzed using IBM SPSS Statistics version 25.0 for Windows (IBM, Chicago, IL, USA) and JASP version 0.19.1.0 (Boston, MA, USA). The significance level was set at 0.05.

Additionally, effect size was calculated using the rank-biserial correlation to quantify the magnitude of differences between pretest and posttest scores. The results were interpreted as follows: values around 0.1 indicate a small effect, values around 0.3 reflect a medium effect, and values of 0.5 or higher represent a large effect. Furthermore, 95% confidence intervals were reported. No methods for imputing missing data were applied, as the analysis was performed using the complete available dataset. Finally, based on previous evidence of sex-based differences in psychological responses to physical activity and meditation, analyses were also stratified by sex to explore potential differential effects.

## 3. Results

### 3.1. Participants Characteristics

Table 1 presents the sociodemographic characteristics of the participants, broken down by sex and group (CG and IG). The variables include average age, academic performance, receipt of financial aid, type of habitual residence, and smoking habits.

Regarding age, participants reported a mean age, indicating that the sample had a relatively homogeneous age distribution. The vast majority reported an academic performance at level B, while higher grades (A) were less common.

In terms of residence type, family homes and shared student apartments were the most prevalent, while student dormitories and privately owned homes were less frequently reported.

Concerning smoking habits, most participants indicated that they do not smoke, alt-hough differences between groups and sex were observed, which may be relevant for fur-ther analysis.

Table 2 presents pre-test values to assess homogeneity between the control and experimental groups, broken down by gender. Statistically significant initial differences were identified in vigor mood state among men (CG: 11.24 ± 3.84 vs. IG: 9.98 ± 2.52; *p* = 0.019) and in fatigue among women (CG: 8.45 ± 3.17 vs. IG: 6.22 ± 3.13; *p* = 0.005), which should be considered when interpreting the results. Overall, the groups demonstrated homogeneity in most of the evaluated variables, validating their initial comparability and allowing subsequent effects to be attributed to the intervention program.

### 3.2. Intervention Effects

Table 3 presents the pre-test and post-test values for the evaluated variables, as well as the observed differences for women. Among the most notable results, the intervention program had a significant impact on resilience in the IG, with an improvement of 6.31 ± 3.87 (*p* = 0.001), compared to the CG, which showed minimal change. Additionally, the IG exhibited a reduction in depressive symptoms, assessed both through the DASS-21 (−3.28 ± 8.79) and the POMS (−0.50 ± 4.88). In contrast, the CG showed an increase in depression scores compared to the pre-test measurements. Regarding sleep duration, the IG experienced a slight increase (0.05 ± 0.46), whereas the CG showed a decrease (0.37 ± 0.49). On the other hand, no statistically significant results (*p* > 0.05) were found in psychological distress-related variables such as anxiety and stress, nor in self-esteem, sedentary hours, combined physical activity, personality traits, or anger and tension states.

Table 4 presents the pre-test and post-test values of the variables for men. The IG showed a significant improvement in resilience (9.37 ± 12.63; *p* < 0.001) after the intervention, while the CG experienced a slight decrease (-0.92 ± 5.19). Regarding depressive symptoms, a significant reduction was observed in the IG (-3.57 ± 5.12; *p* = 0.004), whereas the CG showed a slight increase (1.52 ± 4.41). Additionally, the IG experienced a significant increase in sleep duration (0.55 ± 0.33; *p* = 0.031), whereas no relevant changes were observed in the CG. The IG showed a significant decrease in fatigue (–1.54 ± 4.86; *p* < 0.001) and depression (–5.12 ± 4.25; *p* < 0.001), measured by the POMS questionnaire. Likewise, a significant reduction in tension levels was observed in the IG (–1.76 ± 4.79; *p* = 0.008), whereas changes in the CG were not significant. On the other hand, no significant differences were found in variables such as anxiety, stress, self-esteem, combined PA, sedentary hours, or personality traits.

## 4. Discussion

The present study evaluated the effect of the Healthy Pills intervention, which focuses on physical activities and meditation, designed to improve psychological well-being in university students. It was hypothesized that students in the IG would exhibit significant improvements in resilience, self-esteem, and positive mood states, as well as significant reductions in psychological distress, including depression, anxiety, and stress, compared to the CG. The results identified significant areas of improvement in the IG, particularly in resilience, depressive symptoms, and sleep duration, which supports the hypothesis. However, the hypothesis was partially met, as significant improvements were not observed in self-esteem, anxiety, stress, personality traits, and certain mood states.

The intervention significantly enhanced resilience in both men and women, with a more pronounced impact on males. This could be explained by the fact that men generally report higher resilience levels, influenced by cultural expectations that promote independence and problem-solving (Luibl et al., 2021; Rayani et al., 2024; de Andrade et al., 2024). Conversely, women often face greater social and academic pressures, increasing their vulnerability to stress and lowering their perceived resilience (Gefen & Fish, 2019; Montolio & Taberner, 2021). Despite these differences, the program strengthened adaptation and recovery capacities in both groups, demonstrating its effectiveness in a university setting.

Meditation and PA play a crucial role in fostering resilience. Previous studies indicate that MI promotes well-being and enhances resilience in university students (Galante et al., 2018). Practices such as yoga, which integrate breathing techniques and conscious body movement, have shown significant benefits in emotional resilience (Castellote-Caballero et al., 2024). Moreover, meditation increases hope, a key factor in resilience (Muñoz et al., 2018) and leads to sustained improvements in mental and physical health over time (Haglund et al., 2007; Stallard & Buck, 2013). Meanwhile, PA strengthens resilience by improving self-regulation and neural connections (Belcher et al., 2021), protecting against cognitive decline and neurological diseases, and enhancing adaptability to challenges (Arida & Teixeira-Machado, 2021).

The intervention also led to a significant reduction in depressive symptoms within the IG, supporting the effectiveness of aerobic exercise and strength training as both therapeutic and complementary treatments for depression (Kvam et al., 2016). Additionally, MI, which integrates body-mind techniques, regulates depression through mechanisms such as endorphin release and modulation of the hypothalamic-pituitary-adrenal axis (Mahindru et al., 2023). However, their effectiveness depends on sustained commitment, which limits the impact of short-term interventions in achieving long-lasting changes. This is particularly relevant in university populations, where it is essential to distinguish between therapeutic and preventive approaches. Short-term interventions often provide immediate benefits but lack strong evidence supporting their long-term sustainability in psychological and emotional variables (Koopman et al., 2017). In contrast, therapeutic approaches tend to produce more lasting effects, especially within the first six months (Werner-Seidler et al., 2017). Combining both strategies could maximize both short-term and long-term benefits.

Sleep duration also showed significant improvements in the IG for both men and women, aligning with studies that link moderate-to-vigorous intensity exercise with better sleep quality (Gerber et al., 2014). The inclusion of both low- and high-intensity activities in the intervention may have contributed to these results, in contrast to research focused solely on low-intensity exercises, which found no significant changes (Choi et al., 2018). Furthermore, evidence suggests that both acute sessions and regular PA practice positively impact sleep parameters (PAGAC, 2018), highlighting the importance of varying intensities to optimize sleep quality and duration.

Participants also experienced positive changes in mood, particularly in reduced ten-sion, anger, and fatigue, with more noticeable effects in men. These findings reinforce previous evidence that aerobic PA enhances vigor and vitality and reduces negative emotional states. Additionally, cross-sectional studies have identified inverse correlations between total PA and factors such as hostility, fatigue, and mood disorders, as well as positive correlations between PA and vigor (Evangelista et al., 2014; Legey et al., 2017). However, the lack of longitudinal studies examining the effects of PA or meditation on mood in healthy university populations limits the ability to draw solid conclusions regarding long-term benefits.

Despite these positive results, no statistically significant differences were found in variables such as anxiety, stress, self-esteem, personality traits, sedentary hours, PA levels, and certain mood states. This is noteworthy, considering that multiple studies have shown that both PA and meditation can reduce anxiety and stress (Fazia et al., 2023; Margulis et al., 2023), improve self-esteem (Sani et al., 2016; Tripathi et al., 2024), positively influence mood (Fennell et al., 2022), modify personality traits (Müller, 2023), and promote changes in sedentary behavior (Teno et al., 2024). A possible explanation for these findings is the brief duration of the intervention. Significant changes in relatively stable psychological dimensions often require longer pro-grams that allow participants to consolidate healthy habits such as regular PA and meditation. Repetition is key to automatizing these behaviors and reducing the effort needed to maintain them. In short-term interventions, initial motivation may decline before habits become firmly established, particularly when participants face psychological distress, fatigue, or self-regulation challenges.

Another factor that may have influenced these results is that the intervention did not meet the PA minimums established by international health organizations (WHO, 2020; PAGAC, 2018), which recommend at least 150 min per week of moderate activity or 75 min of vigorous activity, along with muscle-strengthening exercises twice a week. Meeting these guidelines is essential for improving physical and psychological well-being, and integrating meditation may amplify these benefits.

Ekelund et al. (2019) demonstrated that even brief PA sessions provide physical and mental benefits, while a meta-analysis of 48 studies highlighted a dose-response relationship, showing that achieving 5000 MET-minutes per week significantly reduces the risk of mortality from non-communicable diseases (Blond et al., 2020). However, the specific dose-response relationship between PA and mental and cognitive health outcomes remains unclear (PAGAC, 2018), underscoring the need for further research in this area.

### 4.1. Limitations

This intervention presents several limitations that should be considered. First, a non-randomized quasi-experimental design was used, which may have introduced biases due to the lack of randomization in the assignment of participants to the control and intervention groups. Additionally, complete blinding was not possible due to the practical nature of the activities performed (meditation and physical conditioning). However, measures were taken to minimize this bias, such as blinding data analysts and outcome assessors, conducting a baseline comparison between groups to ensure initial homogeneity, and following standardized protocols during the intervention.

Furthermore, the design of the ‘Healthy Pill’ intervention included two distinct approaches—PA and meditation—which have separate effects on psychological well-being. Since the intervention combined both approaches, it was not possible to isolate the specific contributions of each component. Further research is needed to evaluate the individual effects of physical activity and meditation separately, as well as their combined impact. This would help to better understand how each intervention contributes to mental health outcomes and whether both are necessary to achieve the desired effects.

The 12-week duration of the intervention may have been insufficient to observe significant and sustained changes in more stable psychological and emotional variables. In line with existing research, interventions should ideally be prolonged for more than 12 weeks, with a frequency of at least three sessions per week, to observe more significant and lasting effects on psychological well-being (Rosales-Ricardo & Ferreira, 2022; Xiao et al., 2021). While the exact parameters may vary, there is a consensus in the literature on the importance of these factors for achieving optimal outcomes (PAGAC, 2018). Furthermore, all variables were assessed using self-report questionnaires, which may have introduced biases associated with participants’ subjective perception. Although the Cronbach’s alpha for the IPAQ (0.73–0.77) falls within the range reported in previous re-search (0.71–0.89; Dinger et al., 2006), it does not reach the optimal reliability coefficient (>0.80) suggested for comparing group differences (Viladrich et al., 2017).

A limitation of the analysis is the exclusion criterion, which removed students who attended less than 90% of the intervention sessions. We did not perform additional analyses such as Last Observation Carried Forward (LOCF) or mixed models to incorporate incomplete data. The analyses presented in the study are based only on the data from students who completed the intervention. While we acknowledge that including incomplete data might have provided a more comprehensive analysis, the focus of this study was on the results from those who fully participated according to the exclusion criterion.

Additionally, the study did not perform any formal analysis comparing students who completed the intervention with those excluded from the analysis. This may impact the generalizability of the findings, and future studies could benefit from including all participants or performing analyses with missing data imputation techniques. Moreover, the implementation of the intervention within the framework of a university course posed structural limitations. Logistical and academic constraints prevented the formation of multiple intervention groups or the use of a randomized controlled trial design. Consequently, the structure of the course partially dictated the study design, which may limit the generalizability of the findings and the strength of the conclusions.

Another relevant factor is the influence of external effects, such as academic periods coinciding with the intervention (holidays or increased workload at the end of the semester), which may have biased mental health behaviors and affected adherence. Moreover, no extended follow-up was conducted after the intervention, limiting the evaluation of sustained effects on key variables such as resilience, sleep, and mood states.

It is interesting to note that all the subjects of this study were physical education majors. As these students may be more physically active than the average student population, the results may not be fully generalizable to all college students. That some of the variables, like anxiety and self-esteem, yielded non-significant findings could be due to a ceiling effect. Since the participants in this study were already relatively high on PA levels and psychological well-being, perhaps the intervention did not yield large gains in these domains because the baseline levels were moderate to high.

### 4.2. Recommendations for Future Research

Future studies should explore strategies to optimize interventions aimed at improving mental health and well-being in university populations. It is essential to design research with larger and more diverse samples, ensuring gender balance, as well as to implement longer intervention and follow-up periods to assess long-term effects. Additionally, minimizing seasonal effects, such as academic breaks or increased workload at the end of the semester, would help reduce potential biases in the results.

In this context, future interventions could integrate in-person and digital approaches, leveraging the widespread use of the Internet and smartphones among university students. Tools such as mobile applications, web-based platforms, and exergames could enhance accessibility and improve adherence. Strategies like sending daily motivational messages tailored to the academic environment and individual characteristics could further encourage PA and sustain commitment, maximizing long-term impact (Figueroa et al., 2022). Moreover, investigating the impact of hybrid approaches on adherence, well-being, and the sustainability of healthy habits in this population would be of particular interest.

Further, a qualitative evaluation of future intervention would prove to be highly beneficial to comprehend participants’ subjective attitudes and experiences, complementing the quantitative findings. The evaluation would be capable of uncovering factors influencing participation, barriers to participation, and the individual impact of the intervention, providing a better overall picture of its efficacy and scope for improvement.

Despite these limitations, this study makes a significant contribution by exploring interventions aimed at improving mental health and well-being among university students in Spain, adding longitudinal data to a relatively underexplored area.

## 5. Conclusions

The present study explored the effects of the Healthy Pills intervention, which integrates PA and meditation, on the psychological well-being of university students. The results demonstrated significant improvements in resilience, depressive symptoms, and sleep duration, highlighting its potential to address the emotional and psychological challenges faced by this population.

Despite certain limitations, such as the intervention’s duration, reliance on self-reports, and lack of long-term follow-up, the findings reinforce the effectiveness of combining mind-body practices with PA to enhance mental health. Additionally, integrating digital technologies, such as mobile applications and exergames, could improve accessibility and adherence, further enhancing the impact and sustainability of these interventions. To optimize their effectiveness, it is crucial to differentiate between preventive and therapeutic approaches based on the specific characteristics of students.

In summary, Healthy Pills offers a practical and adaptable strategy with strong potential to foster psychological well-being among university students. Given the scarcity of research on this topic in Spain, this study provides valuable insights and serves as a foundation for future research and interventions aimed at improving mental health in this population.

## Figures and Tables

**Table 1:** Characteristics of the sample.

Characteristics		CG		IG	
		Male (*n* = 24)	Female (*n* = 30)	Male (*n* = 46)	Female (*n* = 36)
Age (years) ± SD		21.7 ± 1.1	22.1 ± 1.7	21.8 ± 1.2	21.3 ± 0.5
Grade Point Average	B “Very good” *n* (%)	23 (95.8)	28 (93.3)	42 (91.3)	33 (91.7)
	A “Excellent” *n* (%)	1 (4.2)	2 (6.7)	4 (8.7)	3 (8.3)
Financial Grant for Academic Year	Yes *n* (%)	13 (54.2)	20 (66.7)	26 (56.5)	18 (50.0)
	No *n* (%)	11 (45.8)	10 (33.3)	20 (43.5)	18 (50.0)
Place of Residence During Academic Year	Student Shared Flat *n* (%)	11 (45.8)	19 (63.3)	25 (54.3)	21 (58.3)
	Family Home *n* (%)	10 (41.7)	9 (30.0)	21 (45.7)	13 (36.1)
	Student residence *n* (%)	1 (4.2)	1 (3.3)	-	2 (5.6)
	Own home *n* (%)	2 (8.3)	1 (3.3)	-	-
Self-reported Smoker	Yes *n* (%)	6 (25.0)	3 (10.0)	12 (26.1)	7 (19.4)
	No *n* (%)	18 (75.0)	27 (90.0)	34 (73.9)	29 (80.6)

Note: CG: control group; IG: intervention group.

**Table 2:** Homogeneity test of variables between CG and IG by sex.

	Males (*n* = 71)			Females (*n* = 65)		
Outcomes	CG (*n* = 25)	IG (*n* = 46)	*p* Value	CG (*n* = 29)	IG (*n* = 36)	*p* Value
	Mean ± SD	Mean ± SD		Mean ± SD	Mean ± SD	
Depression (DASS-21)	10.72 ± 8.28	10.83 ± 10.10	0.641	13.66 ± 8.38	13.78 ± 9.69	0.806
Anxiety	10.24 ± 8.09	8.83 ± 7.91	0.336	10.00 ± 7.13	13.28 ± 7.90	0.089
Stress	15.52 ± 7.29	15.78 ± 7.69	0.586	17.59 ± 8.36	18.56 ± 9.07	0.801
Resilience	69.56 ± 10.28	72.57 ± 11.81	0.323	74.03 ± 10.50	72.86 ± 10.06	0.369
Self-esteem	30.96 ± 4.81	33.17 ± 5.26	0.055	30.41 ± 4.79	30.50 ± 9.69	0.751
Anger	7.00 ± 5.11	8.20 ± 3.52	0.380	7.55 ± 5.22	5.28 ± 4.10	0.076
Vigor	11.24 ± 3.84	9.98 ± 2.52	0.019	10.31 ± 2.75	9.67 ± 2.89	0.384
Fatigue	7.04 ± 3.95	6.83 ± 2.55	0.549	8.45 ± 3.17	6.22 ± 3.13	0.005
Tension	9.88 ± 5.88	9.09 ± 4.71	0.530	10.66 ± 4.09	9.25 ± 4.44	0.080
Depression (POMS)	6.44 ± 4.86	6.07 ± 3.13	0.933	6.79 ± 4.10	5.14 ± 3.09	0.150
Extraversion	25.48 ± 2.33	25.70 ± 1.59	0.539	25.28 ± 2.09	25.36 ± 1.92	0.758
Agreeableness	26.28 ± 2.74	27.28 ± 3.18	0.071	27.17 ± 2.58	26.56 ± 2.98	0.348
Conscientiousness	26.60 ± 3.51	25.09 ± 3.87	0.087	25.76 ± 4.62	26.97 ± 3.79	0.284
Neuroticism	24.56 ± 2.24	24.67 ± 2.47	0.898	24.86 ± 2.60	25.78 ± 2.75	0.279
Openness	28.88 ± 3.63	29.87 ± 3.11	0.284	29.83 ± 2.56	30.31 ± 2.94	0.563
Combined PA ^1^	4621.56 ± 1430.94	4476.13 ± 1594.57	0.691	4415.28 ± 1754.54	4132.93 ± 1454.56	0.746
Sedentary Behavior ^2^	4.84 ± 1.77	4.50 ± 1.55	0.560	5.10 ± 1.83	4.42 ± 1.75	0.127
Sleep ^2^	6.86 ± 0.29	6.48 ± 0.19	0.580	7.04 ± 0.40	7.04 ± 0.25	0.550

Note: CG: control group; IG: intervention group; PA: physical activity; ^1^ (Measured in MET-min/week); ^2^ (measured in hours).

**Table 3:** Effects of the intervention program on university female students.

FEMALES		Pretest Mean ± SD	Posttest Mean ± SD	Difference (Posttest- Pretest) Mean ± SD	*p* Value	Rank-Biserial Correlation		IC 95% Lower; Upper
Depression– DASS-21	CG	13.66 ± 8.38	14.69 ± 8.82	1.03 ± 8.43	0.021	−0.072	<small>	[−0.342, 0.209]
	IG	13.78 ± 9.69	10.50 ± 6.82 *	−3.28 ± 8.79				
Anxiety	CG	10.00 ± 7.13	8.76 ± 7.06	1.24 ± 7.34	0.117	−0.226	<small>	[−0.473, 0.054]
	IG	13.28 ± 7.90	9.17 ± 6.75 **	−4.11 ± 6.71				
Stress	CG	17.59 ± 8.36	17.10 ± 8.38	−0.48 ± 7.61	0.418	−0.117	<small>	[−0.382, 0.166]
	IG	18.56 ± 9.07	15.00 ± 6.51 *	−3.56 ± 8.21				
Resilience	CG	74.03 ± 10.50	74.41 ± 11.28	0.38 ± 6.57	0.001	0.468	<large>	[0.220, 0.660]
	IG	72.86 ± 10.06	79.17 ± 8.49 ***	6.31 ± 7.83				
Self-esteem	CG	30.41 ± 4.78	30.55 ± 5.51	0.14 ± 3.56	0.131	0.218	<small>	[−0.062, 0.467]
	IG	30.50 ± 4.40	31.58 ± 4.46	1.08 ± 3.97				
Openness	CG	29.83 ± 2.56	30.00 ± 2.42	0.17 ± 2.52	0.155	−0.204	<small>	[−0.455, 0.077]
	IG	30.31 ± 2.94	29.83 ± 3.13	−0.47 ± 2.44				
Anger	CG	7.55 ± 5.22	7.97 ± 6.24	0.41 ± 7.19	0.517	0.047	<small>	[−0.233, 0.320]
	IG	5.28 ± 4.10	3.69 ± 3.45	−1.58 ± 5.88				
Vigor	CG	10.31 ± 2.75	12.21 ± 3.40	1.89 ± 3.09	0.058	0.000	<small>	[−0.277, 0.277]
	IG	9.67 ± 2.90	9.89 ± 3.35 **	0.22 ± 3.43				
Fatigue	CG	8.45 ± 3.17	11.48 ± 4.50	3.03 ± 5.16	0.012	−0.092	<small>	[−0.360, 0.190]
	IG	6.22 ± 3.13	5.56 ± 4.21 **	−0.66 ± 5.50				
Tension	CG	10.66 ± 4.09	10.24 ± 4.97	−0.41 ± 4.82	0.372	0.151	<small>	[−0.131, 0.411]
	IG	9.25 ± 4.44	7.81 ± 2.92	−1.44 ± 5.09				
Depression- POMS	CG	6.79 ± 4.10	11.34 ± 5.23 ***	4.55 ± 5.09	0.001	−0.119	<small>	[−0.383, 0.164]
	IG	5.14 ± 3.09	5.64 ± 3.97	−0.50 ± 4.88				
Extraversion	CG	25.28 ± 2.08	25.00 ± 1.95	−0.28 ± 7.61	0.464	0.105	<small>	[−0.177, 0.372]
	IG	25.36 ± 1.91	25.61 ± 2.34	0.25 ± 2.41				
Agreeableness	CG	27.17 ± 2.58	26.83 ± 2.17	−0.34 ± 2.17	0.200	0.183	<small>	[−0.099, 0.438]
	IG	26.56 ± 2.98	26.83 ± 2.86	0.28 ± 2.09				
Conscientiousness	CG	25.76 ± 4.62	26.24 ± 4.05	0.48 ± 2.26	0.179	−0.192	<small>	[−0.445, 0.090]
	IG	26.97 ± 3.79	26.50 ± 3.37	−0.47 ± 2.40				
Neuroticism	CG	24.86 ± 2.60	24.76 ± 2.29	−0.10 ± 2.06	0.485	−0.101	<small>	[−0.367, 0.182]
	IG	25.78 ± 2.75	25.06 ± 2.81	−0.72 ± 2.12				
Combined PA ^1^	CG	4415.28 ± 1754.54	4378.24 ± 1872.96	−37.03 ± 1372.88	0.889	−0.020	<small>	[−0.296, 0.258]
	IG	4132.93 ± 1454.56	4163.03 ± 1403.08	30.09 ± 1486.20				
Sedentary Behavior ^2^	CG	9.00 ± 5.10	8.00 ± 4.66	−0.44 ± 2.04	0.840	0.029	<small>	[−0.250, 0.303]
	IG	7.00 ± 4.42	6.00 ± 4.22	−0.19 ± 1.56				
Sleep ^2^	CG	7.04 ± 0.40	6.67 ± 0.28	−0.37 ± 0.49	0.012	−0.340	<medium>	[−0.500, −0.190]
	IG	7.04 ± 0.25	7.09 ± 0.39	0.05 ± 0.46				

Note: GC: control group; IG: intervention group; PA: physical activity; * *p* < 0.05; ** *p* < 0.01; *** *p* < 0.001; small (rank-biserial correlation ≈ 0.1); medium (rank-biserial correlation ≈ 0.3); large (rank-biserial correlation ≈ 0.5); ^1^ (measured in MET-min/week); ^2^ (measured in hours).

**Table 4:** Effects of the intervention program on university male students.

MALES		Pretest Mean ± SD	Posttest Mean ± SD	Difference (Posttest- Pretest) Mean ± SD	*p* Value	Rank-Biserial Correlation		IC 95% Lower; Upper
Depression– DASS-21	CG	10.72 ± 8.28	12.24 ± 8.89	1.52 ± 4.41	0.004	−0.406	<large>	[−0.613, −0.147]
	IG	10.83 ± 10.09	7.26 ± 7.49 **	−3.57 ± 8.71				
Anxiety	CG	10.24 ± 8.08	9.84 ± 7.18	−0.40 ± 5.32	0.464	−0.103	<small>	[−0.368, 0.178]
	IG	8.83 ± 7.90	5.91 ± 4.36 *	−2.91 ± 7.00				
Stress	CG	15.52 ± 7.28	14.88 ± 7.14	−0.64 ± 6.40	0.109	−0.229	<small>	[−0.475, 0.050]
	IG	15.78 ± 7.68	12.17 ± 7.54 **	−3.61 ± 7.39				
Resilience	CG	69.56 ± 10.28	68.64 ± 9.14	−0.92 ± 5.19	<0.001	0.597	<large>	[0.384, 0.749]
	IG	72.57 ± 11.81	81.93 ± 11.67 ***	9.37 ± 12.63				
Self-esteem	CG	30.96 ± 4.80	31.52 ± 4.67	0.56 ± 2.50	0.481	0.101	<small>	[−0.180, 0.366]
	IG	33.17 ± 5.25	34.87 ± 4.42 *	1.70 ± 5.06				
Anger	CG	7.00 ± 5.10	9.33 ± 6.56	2.62 ± 7.75	<0.001	−0.409	<large>	[−0.618, −0.147]
	IG	8.20 ± 3.52	3.93 ± 3.19 ***	−4.26 ± 4.49				
Vigor	CG	11.24 ± 3.84	10.72 ± 4.36	−0.52 ± 2.58	<0.001	0.752	<large>	[0.601, 0.851]
	IG	9.98 ± 2.52	12.98 ± 2.52 ***	3.00 ± 3.04				
Fatigue	CG	7.04 ± 3.95	10.32 ± 5.67 ***	3.28 ± 3.29	<0.001	−0.283	<medium>	[−0.519, −0.008]
	IG	6.83 ± 2.55	5.28 ± 4.23 *	−1.54 ± 4.86				
Tension	CG	9.88 ± 5.87	11.20 ± 6.00	1.32 ± 3.64	0.008	0.043	<small>	[−0.236, 0.315]
	IG	9.09 ± 4.72	7.33 ± 2.33 *	−1.76 ± 4.79				
Depression-POMS	CG	6.44 ± 4.85	11.56 ± 6.96 ***	5.12 ± 4.25	<0.001	−0.222	<small>	[−0.469, 0.058]
	IG	6.07 ± 3.13	5.35 ± 2.88	−0.72 ± 4.17				
Extraversion	CG	25.48 ± 2.33	25.44 ± 2.29	−0.04 ± 1.34	0.478	−0.099	<small>	[−0.365, 0.182]
	IG	25.70 ± 1.59	25.35 ± 1.49	−0.35 ± 1.86				
Agreeableness	CG	26.28 ± 2.74	26.68 ± 2.70	0.40 ± 1.66	0.844	0.028	<small>	[−0.250, 0.301]
	IG	27.28 ± 3.18	27.52 ± 3.22	−0.20 ± 2.73				
Conscientiousness	CG	26.60 ± 3.51	26.32 ± 3.52	−0.28 ± 2.34	0.774	−0.041	<small>	[−0.313, 0.238]
	IG	25.09 ± 3.87	24.89 ± 4.07	0.20 ± 2.73				
Neuroticism	CG	24.56 ± 2.23	25.00 ± 1.68	0.44 ± 1.61	0.306	−0.144	<small>	[−0.404, 0.137]
	IG	24.67 ± 2.47	24.33 ± 2.38	−0.35 ± 2.11				
Openness	CG	28.88 ± 3.63	28.84 ± 4.17	−0.40 ± 2.67	0.456	−0.106	<small>	[−0.371, 0.175]
	IG	29.87 ± 3.11	29.76 ± 3.41	−0.11 ± 2.91				
Combined Physical Activity ^1^	CG	4621.56 ± 1430.94	4690.04 ± 1513.96	68.480 ± 1275.71	0.632	0.069	<small>	[−0.211, 0.338]
	IG	4476.13 ± 1594.57	4580.58 ± 1643.93	104.45 ± 1378.15				
Sedentary Behavior ^2^	CG	4.84 ± 1.77	4.84 ± 1.21	0.00 ± 1.84	0.409	−0.116	<small>	[−0.379, 0.165]
	IG	4.50 ± 1.55	4.11 ± 1.59	−0.39 ± 1.39				
Sleep ^2^	CG	6.86 ± 0.29	6.81 ± 0.15	−0.05 ± 0.33	0.031	0.440	<large>	[0.280; 0.590]
	IG	6.48 ± 0.19	6.85 ± 0.31	0.37 ± 0.36				

Note: CG: control group; IG: intervention group; PA: physical activity; * *p* < 0.05; ** *p* < 0.01; *** *p* < 0.001; small (rank-biserial correlation ≈ 0.1); medium (rank-biserial correlation ≈ 0.3); large (rank-biserial correlation ≈ 0.5); ^1^ (measured in MET-min/week); ^2^ (measured in hours).

## Data Availability

The data supporting the reported results are available at reasonable request from the corresponding author.

## Supplementary Materials

The following supporting information can be downloaded at: https://www.mdpi.com/article/10.3390/bs15040549/s1, Figure S1: Summarize of intervention; Table S1: TREND Statement Checklist.

## Abbreviations

The following abbreviations are used in this manuscript:

CG	Control Group
GDPR	General Data Protection Regulation
IG	Intervention Group
LOCF	Last Observation Carried Forward
MI	Meditation-based intervention
MH	Mental Health
PA	Physical Activity
PAGAC	Physical Activity Guidelines Advisory Committee
TREND	Transparent Reporting of Evaluations with Nonrandomized Designs
WHO	World Health Organization
WMH-ICS	World Mental Health International College Student
